# A multi-factorial analysis of response to warfarin in a UK prospective cohort

**DOI:** 10.1186/s13073-015-0255-y

**Published:** 2016-01-06

**Authors:** Stephane Bourgeois, Andrea Jorgensen, Eunice J. Zhang, Anita Hanson, Matthew S. Gillman, Suzannah Bumpstead, Cheng Hock Toh, Paula Williamson, Ann K. Daly, Farhad Kamali, Panos Deloukas, Munir Pirmohamed

**Affiliations:** Wellcome Trust Sanger Institute, Genome Campus, Hinxton, Cambridgeshire CB10 1SA UK; University of Liverpool, Liverpool, Merseyside L69 3GE UK; Newcastle University, Newcastle upon Tyne, UK; Royal Liverpool and Broadgreen University Hospital NHS Trust, Liverpool, L7 8XP UK; William Harvey Research Institute, Barts and The London School of Medicine and Dentistry, Queen Mary University of London, Charterhouse Square, London, EC1M 6BQ UK; The Wolfson Centre for Personalised Medicine, Institute of Translational Medicine, University of Liverpool, Block A: Waterhouse Building, 1-5 Brownlow Street, Liverpool, L69 3GL UK

## Abstract

**Background:**

Warfarin is the most widely used oral anticoagulant worldwide, but it has a narrow therapeutic index which necessitates constant monitoring of anticoagulation response. Previous genome-wide studies have focused on identifying factors explaining variance in stable dose, but have not explored the initial patient response to warfarin, and a wider range of clinical and biochemical factors affecting both initial and stable dosing with warfarin.

**Methods:**

A prospective cohort of 711 patients starting warfarin was followed up for 6 months with analyses focusing on both non-genetic and genetic factors. The outcome measures used were mean weekly warfarin dose (MWD), stable mean weekly dose (SMWD) and international normalised ratio (INR) > 4 during the first week. Samples were genotyped on the Illumina Human610-Quad chip. Statistical analyses were performed using Plink and R.

**Results:**

VKORC1 and CYP2C9 were the major genetic determinants of warfarin MWD and SMWD, with CYP4F2 having a smaller effect. Age, height, weight, cigarette smoking and interacting medications accounted for less than 20 % of the variance. Our multifactorial analysis explained 57.89 % and 56.97 % of the variation for MWD and SMWD, respectively. Genotypes for VKORC1 and CYP2C9*3, age, height and weight, as well as other clinical factors such as alcohol consumption, loading dose and concomitant drugs were important for the initial INR response to warfarin. In a small subset of patients for whom data were available, levels of the coagulation factors VII and IX (highly correlated) also played a role.

**Conclusion:**

Our multifactorial analysis in a prospectively recruited cohort has shown that multiple factors, genetic and clinical, are important in determining the response to warfarin. VKORC1 and CYP2C9 genetic polymorphisms are the most important determinants of warfarin dosing, and it is highly unlikely that other common variants of clinical importance influencing warfarin dosage will be found. Both VKORC1 and CYP2C9*3 are important determinants of the initial INR response to warfarin. Other novel variants, which did not reach genome-wide significance, were identified for the different outcome measures, but need replication.

**Electronic supplementary material:**

The online version of this article (doi:10.1186/s13073-015-0255-y) contains supplementary material, which is available to authorized users.

## Background

Warfarin is the most widely used oral anticoagulant worldwide for the treatment of thromboembolic disorders [1]. The wide inter-individual variability in warfarin dose requirement, and its narrow therapeutic index makes the outcome of treatment difficult to predict; under-anticoagulation can predispose patients to thrombosis, while over-anticoagulation increases the risk of bleeding [2]. A number of interventions have been used to improve the accuracy of warfarin dosing including home monitoring [3], computer-based dosing [4], different dosing algorithms [5] and more intensive monitoring [6]. However, despite these measures, accurate warfarin dosing remains difficult to achieve. Warfarin appears in the top three of most epidemiological surveys of adverse reactions causing hospital admission [7]. There is thus a need to improve the safety of warfarin.

Genetic factors are known to predict warfarin dose requirements - observational studies have shown that variation in *CYP2C9* and *VKORC1*, together with body mass index and age, account for about 50 % of the variation in warfarin daily dose requirements [8]. Many dosing algorithms incorporating both genetic and clinical factors for predicting warfarin doses during initiation and maintenance phases of therapy have been developed [9]. The FDA has changed the drug label [10] because of the distinct role genetics plays on warfarin dose requirement. We have recently undertaken a randomised controlled trial of genotype-guided dosing versus standard clinical practice [11] - this showed that genotyping prior to warfarin prescription increased the time within therapeutic range of international normalised ratio (INR) between 2.0-3.0 by a mean of 7.0 %. However, the COAG trial [12], conducted in the US, which used a different algorithmic strategy did not show any difference in the time within therapeutic INR range between genotype-guided and clinical dosing algorithms. There are several possible reasons for the discordant findings between the two trials [13].

We cannot exclude the possibility that other factors, either genetic, clinical or biochemical, could improve personalisation of dosing for warfarin, and thereby its efficacy and safety. In order to evaluate this, we have undertaken further analyses of our UK prospective cohort study [14], where the clinical phenotype of each patient has been extensively documented, together with measurement of clinical laboratory tests, and the vitamin-K dependent coagulation factors. In this paper, we report on the results of these analyses of the response to warfarin for both the initiation (first week of dosing) and maintenance (stable anticoagulation) periods. Our aim was to identify additional clinical, biochemical and genetic factors that explain the as yet unexplained 50 % of variation in warfarin dose requirements.

## Methods

### Ethics, consent and permissions

Blood samples, demographic and clinical data from patients initiating warfarin for venous thromboembolism or atrial fibrillation between November 2004 and March 2006 were collected, as described previously [14]. Updated patient demographics for this extended cohort are available in Table 1. The study, which conforms to the Declaration of Helsinki, was approved by the Birmingham South Research Ethics Committee, and each patient provided informed consent to participate in the study.

**Table 1:** Summary statistics for all non-genetic factors investigated and outcomes

	N	Min	First quartile	Median	Mean	Third quartile	Max
Age (years)	702	18.85	61.87	71.83	68.71	77.65	94.55
Height (cm)	708	125.00	160.00	168.00	168.10	176.00	193.00
Weight (kg)	705	30.00	67.00	79.00	79.58	90.00	160.00
Loading dose (mg)	711	3.00	14.00	21.00	19.44	25.00	32.00
Mean weekly dose (mg/week)	612	4.28	19.70	26.94	27.10	35.19	87.24
Stable mean weekly dose (mg/week)	326	3.50	20.98	26.83	27.78	35.05	119.03
Alcohol consumption score	711	0.00	1.00	2.00	3.79	5.00	38.00
Follow-up time (day)	629	2.00	176.00	182.00	164.70	184.00	277.00
Time to INR over 4 (day)	618	2.00	3.00	3.00	4.19	6.00	7.00
Time to stability (day)	629	2.00	22.00	51.00	55.87	79.50	165.00
Stability duration (day)	326	14.00	42.00	70.00	76.36	103.50	212.00
Haemoglobin (10^9^/L)	144	8.50	12.80	13.95	13.98	15.30	17.70
Platelets (10^9^/L)	143	4.00	204.00	242.00	265.10	300.00	874.00
White cell count (10^9^/L)	144	4.00	5.90	6.80	8.61	8.50	181.00
Neutrophils (10^9^/L)	144	1.70	3.38	4.30	4.56	5.33	13.20
Lymphocytes (10^9^/L)	144	0.60	1.40	1.85	2.02	2.20	27.90
Monocytes (10^9^/L)	144	0.20	0.48	0.60	0.58	0.70	1.20
Eosinophils (10^9^/L)	144	0.00	0.10	0.15	0.20	0.20	0.80
Basophils (10^9^/L)	144	0.00	0.00	0.00	0.02	0.00	0.40
Potassium (mmol/L)	154	2.60	3.80	4.10	4.06	4.30	5.10
Chloride (mmol/L)	157	1.00	99.00	101.00	100.40	103.00	113.00
Bicarbonate (mmol/L)	157	19.00	24.00	26.00	25.83	28.00	39.00
Urea (mmol/L)	158	1.50	4.63	5.90	6.57	7.30	27.40
Creatinine (umol/L)	158	1.00	77.00	92.00	102.70	108.80	773.00
Triglycerides (mmol/L)	148	0.30	0.90	1.20	1.71	1.53	41.00
Albumin (g/L)	157	27.00	39.00	41.00	41.25	44.00	74.00
Total protein (g/L)	159	17.00	70.50	74.00	74.18	78.00	100.00
Billirubin (umol/L)	153	1.00	8.00	10.00	11.85	15.00	30.00
ALT (U/L)	156	5.00	17.00	22.00	29.90	33.00	279.00
Alkaline phosphate (U/L)	156	36.00	67.00	79.00	90.69	104.00	336.00
Gamma GT (U/L)	147	2.00	25.00	36.00	53.79	62.00	344.00
Fibrinogen	65	0.64	3.04	3.70	4.93	4.40	76.00
Factor II (%)	142	8.37	83.42	93.91	92.86	101.40	160.50
Factor V (%)	142	42.44	113.90	135.70	137.80	159.70	237.10
Factor VII (%)	142	0.75	80.15	94.94	96.12	112.20	204.80
Factor IX (%)	142	22.75	109.00	119.40	119.20	134.50	231.50
Factor X (%)	142	44.82	83.71	96.24	98.16	110.60	175.00
Protein C (%)	141	15.83	72.70	85.20	86.63	96.83	199.40
Protein S (%)	142	41.76	123.90	147.20	153.70	177.30	294.70

#### Patient follow-up

In this prospective cohort study (EGA accession number EGAS00001001130), all patients received usual clinical care with doses being determined either by the anticoagulant clinic or attending physician. There were four fixed study visits for each patient, the first at the time of initiation of warfarin (index visit), then at 1 week, 8 weeks and 26 weeks of warfarin therapy. The index visit was before, or within 2 days of commencing warfarin, in 58 % of patients, 26 % of index visits were on the third day, and the remainder between 4 and 9 days after starting warfarin. Out of 160 patients for which baseline clotting factor and protein levels were measured, only eight had their index visit after starting warfarin (three on day 1, four on day 2, and one on day 4). To confirm that timing of initiation relative to index visit did not significantly influence baseline clotting factor and protein levels, patients were stratified and these measurements compared between strata; trends in clotting factors relating to index day were also investigated. No significant trends were found, most probably because of the important natural population variation observed in the levels of clotting factors and the small number of patients for which data were collected after warfarin initiation (data available on request). Patients also attended anticoagulant clinic between these four fixed visits as per usual clinical practice, and the total number of INR measurements varied (median number of INRs per patient: 15; range: 1–65).

At the index visit, patient demographics, smoking history, current medications and alcohol intake (assessed using the AUDIT questionnaire [15]) were collected. At all subsequent follow-up visits, any adverse effects, changes in warfarin dose or changes in any other medications since the previous visit were recorded. The list of medications classified as interacting with warfarin is available in Additional file 1 on Sheet 1; the interaction coefficient indicates if the substance reduces (−1) or potentiates (+1) the action of warfarin; amiodarone’s coefficient was set to +2 to reflect its strong effect. All other medications taken by patients and deemed not to have any effect on warfarin dosing are also listed in Additional file 1 on Sheet 2.

### Outcomes

For this GWAS, we used the following outcome measures:

1. Mean weekly dose (MWD): mean dose received weekly during a minimum follow-up time of 14 days post-loading; the loading period, that is, the first 3 days of treatment, was not included in the calculations.
2. Stable mean weekly dose (SMWD): mean weekly dose for at least three consecutive visits where INRs were within the targeted range, spanning a minimum of 14 days and with at least 7 days separating the first and middle INR measurements, and the middle and last one.
3. INR >4.0 in the first week on warfarin.

As the frequency distribution of stable warfarin dose was skewed, the data were normalised by taking square-root of stable dose.

### Genotyping, data calling and automated QC

Samples were assayed on the Illumina Human610-Quad BeadChip using the Infinium HD Super Assay (Illumina, San Diego, CA, USA); beadchips were scanned with an iScan. Intensity data, normalised according to the standard Illumina algorithm, was extracted and genotypes called using Illuminus [16]. Sample call rate was calculated and Illuminus re-run using only the samples with a call rate of at least 90 % (to improve cluster definition).

Samples having a call rate of less than 95 % or having autosomal heterozygosity values in the tail of the distribution were excluded. Chromosome X heterozygosity was used to predict gender (samples with values less than 4 % are predicted as male, those with values over 15 % are predicted as female); this was compared to the gender in the original documentation, and discrepancies resolved or samples excluded. A pairwise comparison was run for all samples using 400 well-spaced, common SNPs to identify duplicate samples. Genotypes for each sample were compared to the molecular fingerprint - a set of 26 markers typed using the Sequenom platform - to eliminate the possibility of arraying errors. Identity by descent (IBD) was calculated for all pairs of samples using PLINK [17], and one sample was excluded from each pair for which Pi hat, the proportion IBD, was superior or equal to 0.1875.

### Imputation

Imputation of genotypes was carried out using IMPUTE V2.1 [18], with the filtered combined set of HapMap 3 release 2 (Feb 2009) and 1000 genomes pilot 1 CEU (March 2010) [19]. Full details are provided in the Additional file 2.

### CNV calling and QC

A suite of Perl and R (2009) scripts were used as a framework to utilise the R package CNVtools, available from http://www.bioconductor.org/packages/release/bioc/html/CNVtools.html [20]. All the steps are detailed in the Additional file 2.

### Kasp genotyping

Genotyping of rs112942398 was performed with a custom KASP™ genotyping assay (LGC Genomics Ltd.) using the 68-62 °C touchdown thermal cycling conditions in accordance with the manufacturer’s instructions. Primer sequences are as follow:

- Primer Allele FAM (G) AATCCCAGCACTTTGGGAGGC,
- Primer Allele HEX (T) GTAATCCCAGCACTTTGGGAGGA, and
- Primer Common GGCTGGATTCGGACCCCTGGA.

Approximately 30 ng genomic DNA was amplified in a 5 μL reaction mixture containing 1× high ROX KASP genotyping master mix and 0.07 μL of primer mix. To improve genotype clustering, the plate was thermally cycled for an extra 10 cycles with an annealing/elongation temperature of 64 °C. End-point FAM and HEX signals were read at 30 °C on an ABI 7900HT Fast Real-Time PCR System (Applied Biosystems). As part of quality control, negative controls (n = 2) containing water instead of DNA and 10 % duplicates were included in the run.

### Statistical analyses

Non-genetic variables used for testing univariately for association with each outcome were age, height, weight, BMI, gender, loading dose, total follow-up time, dosing method (manual or computerised), mean target INR, blood count (haemoglobin, platelets, white cells, neutrophils, basophils, lymphocytes, monocytes, eosinophils), potassium, bicarbonate, chloride, urea, creatinine, triglycerides, albumin, total protein, bilirubin, ALT, alkaline phosphate, gamma GT, fibrinogen, coagulation factors II, V, VII, IX and X, Proteins C and S, current smoking status, number of cigarette smoked per day, ex-smoker status, alcohol consumption, interacting co-medication (binary), non-interacting co-medication (binary), sum of effect of interacting co-medications. The coagulation factors were measured as described by Jorgensen *et al.* [8]. For each variable, either a linear (quantitative outcomes) or logistic (binary outcome) regression was used to test for association with outcome in R, and variables found to be significant univariately (*P* ≤0.05) were included as covariates in the linear or logistic regressions used to test for association between each SNP and outcome in turn, carried out in PLINK. When a SNP was found to be significantly associated with the outcome tested (at genome-wide significance level, *P* ≤5 × 10^−8^), it was added as a covariate to the multiple regression model and each SNP was then re-tested for association with the outcome using this updated model. This process was repeated until no further SNP reached genome-wide significance.

To avoid collinearity, all variables were checked for pairwise correlation using Pearson’s correlation test in R; pairs with a correlation over 0.7 were deemed highly correlated and, in the event that they were found significantly associated with in any of the investigated outcomes, only the one variable with the lowest *P* value was adjusted for when testing for association with the SNPs.

For each outcome, all significantly associated SNPs at genome-wide level, as well as the non-genetic variables found significant univariately were then included together in a multiple regression model in R. Stepwise variable selection was applied to the model to establish a final model. When known variants influencing warfarin dosing (such as *VKORC1 rs9923231*, CYP2C9*2 or *3, and *CYP4F2 rs21*08622) did not reach genome-wide significance, possibly through lack of power, they were added to the stepwise variable selection in order to determine if they would indeed improve the final model.

Manhattan and regional plots were prepared using in-house Python scripts. MWD results were further analysed through the use of IPA (Ingenuity® Systems, www.ingenuity.com). Canonical pathways analysis identified the pathways from the IPA library of canonical pathways that were most significant to the dataset. SNPs considered for canonical pathway analysis had a *P* value lower than 10^−03^.

## Results

### Demographics of patients

The mean age was 69 years (range: 19–95 years) and 55.6 % were men. Patients were treated for atrial fibrillation (AF; 66.0 %), venous thromboembolism (VTE; 24.5 %), cardiovascular disease (comprising ischaemic heart disease, congenital cardiac failure, heart valve disease and other pathologies, total 7.6 %) or other conditions (comprising respiratory disease, cerebrovascular accident/transient ischaemic attack, gastrointestinal disease, neurological disease and other pathologies, total 2.1 %).

### Clinical and biochemical factors

Seven patients failed the genotyping rate threshold, nine failed the heterozygosity criteria, 15 were excluded based on ethnicity using principal component analysis with HapMap3 samples as they did not cluster with samples of European ancestry (data not shown), three had ambiguous gender, three were excluded because of IBD, and four patients decided not to participate in the study, leaving 711 available genotypes for analysis. One hundred patients stopped treatment within the first 2 weeks of treatment, not allowing their inclusion for mean weekly dose calculation, though 13 with appropriate available data were included in INR >4 calculations.

Out of 612 patients with dose and INR information fitting our SMWD outcome, only 326 (53.3 %) reached stability over the follow-up period, which covered up to 277 days. One hundred and nineteen (16.6 %) patients were current smokers (N = 711, mean number of cigarettes = 12.7, sd = 8.9). Out of 625, 116 (18.6 %) patients had an INR >4.0 during the first week of treatment. Out of the 711 patients retained for analysis, 160 had baseline data on full blood count, liver enzymes and coagulation factor levels. Weight and BMI were highly correlated (R^2^ = 0.73), as were gender and height (R^2^ = 0.83), current smoking status and number of cigarettes smoked (R^2^ = 0.79); therefore, only the most significant variable of each pair was kept as a covariate in the regression analyses.

For MWD, age (*P* = 1.20 × 10^−17^), height (*P* = 5.10 × 10^−07^), weight (*P* = 5.02 × 10^−10^), total follow-up time (*P* = 9.41 × 10^−03^), number of cigarettes smoked per day (*P* = 5.34 × 10^−06^), ex-smoker status (*P* = 3.01 × 10^−04^), alcohol consumption (*P* = 2.00 × 10^−04^) as well as the use of interacting co-medications (*P* = 6.30 × 10^−03^), the sum of interactions (*P* = 1.38 × 10^−04^), and the use of medications not classified as interacting (*P* = 2.94 × 10^−02^) were found significant univariately and therefore adjusted for when testing for association with each SNP. MWD increased with height and weight, and decreased with age; it was higher for smokers and increased with the number of cigarettes smoked each day, while it appeared to be lower for ex-smokers in comparison to people who never smoked. MWD was lower in patients taking interacting co-medications, and decreased as the sum of interactions increased, but it also appeared to be lower in patients taking co-medications which are not on the interacting medications list.

In the subset of samples with extra baseline data, age (*P* = 2.06 × 10^−05^), height (*P* = 2.43 × 10^−03^), weight (*P* = 3.58 × 10^−04^), haemoglobin (*P* = 3.64 × 10^−02^), basophils (*P* = 9.20 × 10^−03^), urea (*P* = 4.16 × 10^−02^), ALT (*P* = 4.71 × 10^−02^), Factor IX (*P* = 7.19 × 10^−03^) and current smoking status (*P* = 3.36 × 10^−04^) were found significant univariately and included as covariates in the linear regressions. Though not found univariately significant in this subset of patients, the use of interacting co-medications, the sum of interactions, and the use of medications not classified as interacting were also used as covariates in the linear regressions.

For SMWD, age (*P* = 6.20 × 10^−09^), height (*P* = 1.72 × 10^−05^), weight (*P* = 2.11 × 10^−07^) and the use of interacting co-medications (*P* = 6.62 × 10^−03^) were found significant univariately and adjusted for when testing for association with each SNP. SMWD increased with height and weight, and decreased with age, and was lower in patients taking interacting co-medications.

For INR >4.0 during first week of treatment (INR4), age (*P* = 2.77 × 10^−03^), height (*P* = 1.53 × 10^−02^), weight (*P* = 2.98 × 10^−04^), loading dose (*P* = 7.28 × 10^−07^), alcohol consumption (*P* = 7.76 × 10^−03^) and use of medications (during first week of treatment) not classified as interacting (*P* = 4.28 × 10^−02^) were found significant univariately and included as covariates in the logistic regressions. In addition to these, in the subset of patient with extra baseline data, triglycerides (*P* = 4.97 × 10^−02^), albumin (*P* = 4.23 × 10^−02^) and Factor VII (*P* = 3.82 × 10^−02^) were also found significantly associated with INR4.

### GWAS for MWD and SMWD

**Fig. 1:**
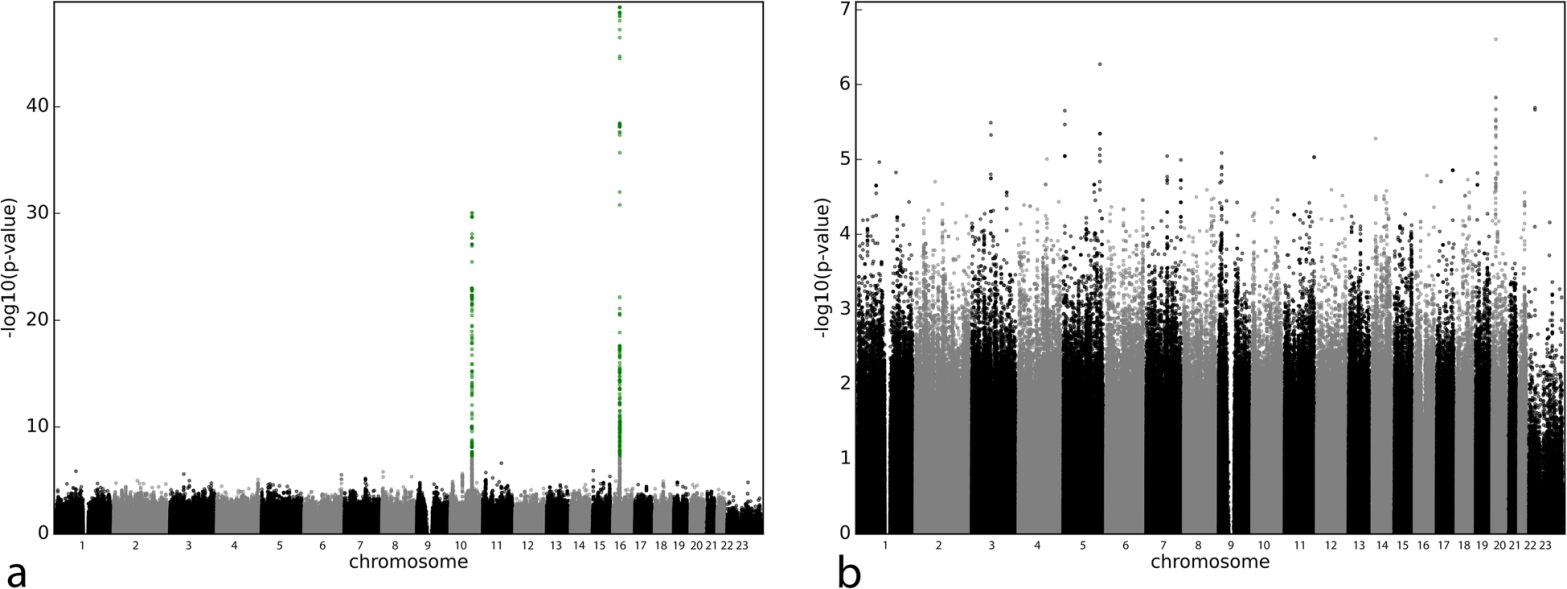
Manhattan plots for multiple regressions on MWD only adjusting for non-genetic factors (**a**) and adjusting for non-genetic and genetic factors (**b**). The vertical axis represents the common logarithm of the *P* value, numbers on the horizontal axis represent the chromosome. Signals below the genome-wide significance threshold of 5 × 10^−08^ are represented in green

For warfarin MWD (Table 2 and Fig. 1a), genome-wide significant signals clustered on chromosome 16 around *VKORC1*, and on chromosome 10 around *CYP2C9*. The top signal in VKORC1 was rs9923231 (*P* = 5.00 × 10^−50^). In CYP2C9, the strongest signal, rs4917639 (*P* = 2.05 × 10^−30^) acts as a composite of CYP2C9*2 and *3 [21]; nevertheless, both *3 (rs1057910, *P* = 1.24 × 10^−23^) and *2 (rs1799853, *P* = 1.65 × 10^−09^) also reached genome-wide significance.

**Table 2:** Top signals from linear regressions on mean weekly dose (MWD)

											MWD (SD) in mg/week		
Variant ID	Chr.	Pos.	Locus	Localisation	Alleles	MAF	Beta (SD)	*P*	HWE	Status	Hom. minor	Het.	Hom. major
rs9923231	16	31015190	VKORC1	Upstream	T/C	0.362	−0.875 (0.05335)	5.00E-50*	1.00	Genotyped	16.8 (6.89)	25.99 (9.64)	35.04 (13.63)
rs4917639	10	96715525	CYP2C9 (composite)	Intronic	C/A	0.208	−0.8334 (0.06864)	2.05E-30*	0.57	Genotyped	14.12 (9.05)	23.77 (9.68)	31.78 (12.92)
rs1057910	10	96731043	CYP2C9 (*3)	Non-synonymous coding	C/A	0.074	−1.146 (0.1094)	1.24E-23*	0.26	Genotyped	7.55 (1.7)	19.08 (7.69)	29.98 (12.66)
rs1799853	10	96692037	CYP2C9 (*2)	Non-synonymous coding	A/G	0.126	−0.59 (0.09629)	1.65E-09*	0.01	Genotyped	16.5 (16.94)	23.96 (10.68)	30.09 (13.06)
rs2876443	20	16850210	OTOR, PCSK2	Intergenic	G/A	0.378	−0.238 (0.04558)	2.48E-07	0.26	Imputed	25 (10.42)	27.78 (12.82)	30.65 (13.2)
5-164557605	5	164557605	U6, AC008415	Intergenic	A/C	0.013	0.9499 (0.1873)	5.34E-07	1.00	Imputed	NaN (NA)	37.45 (19.54)	28.24 (12.47)
rs950353	23	30173133	MAGEB1	Intronic	A/G	0.076	−0.4548 (0.09473)	2.02E-06	0.29	Imputed	25.39 (10.33)	23.97 (10.45)	29.01 (12.98)
rs13188512	5	7557899	ADCY2	Intronic	G/T	0.189	0.2819 (0.05896)	2.21E-06	0.09	Imputed	36.74 (17.71)	30.18 (13.36)	27.37 (12.1)
3-86013074	3	86013074	CADM2	Intronic	T/G	0.054	−0.4597 (0.09774)	3.19E-06	1.00	Imputed	16.39 (6.74)	25.6 (13.31)	28.87 (12.69)
rs6571686	14	34290990	BAZ1A	Downstream	T/A	0.483	−0.2052 (0.04463)	5.22E-06	1.00	Imputed	25.26 (11.44)	29.04 (12.38)	30.14 (14.08)
rs10122258	9	13730846	MPDZ, C9orf146	Intergenic	A/T	0.255	0.2302 (0.05111)	8.11E-06	0.69	Imputed	34.96 (15.49)	28.61 (12.57)	27.66 (12.38)
rs2141221	7	94390435	PPP1R9A	Intronic	T/C	0.460	0.2043 (0.04558)	8.95E-06	0.55	Imputed	31.72 (13.09)	28.43 (12.26)	26.12 (13.07)
rs175811	20	15775473	C20orf133	Intronic	C/T	0.167	0.2633 (0.05882)	9.12E-06	0.50	Imputed	34.15 (13.5)	30.82 (13.7)	27.38 (12.23)
rs1496244	11	133052895	OPCML, SPATA19	Intergenic	G/A	0.348	0.211 (0.04717)	9.27E-06	0.32	Genotyped	29.14 (10.78)	29.42 (13.32)	27.29 (12.62)
4-128288362	4	128288362	Y RNA, INTU	Intergenic	A/G	0.034	0.5585 (0.1252)	9.80E-06	0.56	Imputed	40.07 (NA)	32.7 (12.4)	28.22 (12.78)

*P* values marked with an asterisk were obtained from the regression only adjusting for non-genetic factors, while other *P* values were obtained from a multiple regression after conditioning on rs9923231, rs1799853 and rs1057910. HWE stands for Hardy-Weinberg Equilibrium and gives the *P* value of observed number of heterozygous versus expected number of heterozygous, for each variant

Het., heterozygous; Hom. major, homozygous for the major allele; Hom. minor, homozygous for the minor allele

After adjustment for genetic and non-genetic covariates, no other SNP reached genome-wide significance (Fig. 1b). The previously described signal in CYP4F2, rs2108622 [22, 23] was absent from top hits after multiple regression (Table 2 and Fig. 1b; *P* = 7.70 × 10^−03^), explaining only a further 0.5 % of the MWD variance. Examination of genes at proximity of other signals of interest did not yield any obvious candidates based on gene function. Canonical pathway analysis in IPA, however, using all signals with a *P* value below 10^−03^, highlighted the thrombin signalling pathway, with 12 independent signals with *P* values ranging from 7.8 × 10^−04^ to 2.2 × 10^−06^ (Table 3), and a *P* value of 4.6^−04^for the pathway as a whole. We performed univariate regressions to investigate if any of these 12 variants were linked to coagulation factor levels (data not shown). One variant, rs2298978, appeared significantly associated with Factor IX levels (*P* = 1.26^−03^, Bonferroni threshold: 4.15^−03^).

**Table 3:** Thrombin signalling pathway as reported by IPA

Gene symbol	Entrez gene name	dbSNP	*P* value	Location	Type(s)
ADCY2	Adenylate cyclase 2 (brain)	rs13188512	2.22E-06	Plasma membrane	Enzyme
PRKCE	Protein kinase C, epsilon	rs17034610	1.30E-04	Cytoplasm	Kinase
ITPR1	Inositol 1,4,5-triphosphate receptor, type 1	rs13082052	1.94E-04	Cytoplasm	Ion channel
EGF	Epidermal growth factor	rs2298978	1.97E-04	Extracellular space	Growth factor
ADCY9	Adenylate cyclase 9	rs57119403	3.53E-04	Plasma membrane	Enzyme
PLCB1	Phospholipase C, beta 1 (phosphoinositide-specific)	rs227885	3.79E-04	Cytoplasm	Enzyme
ARHGEF4	Rho guanine nucleotide exchange factor (GEF) 4	rs4322880	3.98E-04	Cytoplasm	Other
PLCE1	Phospholipase C, epsilon 1	rs11187789	6.84E-04	Cytoplasm	Enzyme
EGFR	Epidermal growth factor receptor	rs980653	7.14E-04	Plasma membrane	Kinase
ARHGEF3	Rho guanine nucleotide exchange factor (GEF) 3	rs28641188	7.42E-04	Cytoplasm	Other
PRKCH	Protein kinase C, eta	rs959729	7.46E-04	Cytoplasm	Kinase
MYL9	Myosin, light chain 9, regulatory	rs220076	7.80E-04	Cytoplasm	Other

Canonical pathway analysis, in IPA, of warfarin MWD multivariate analysis, grouped 12 signals below 10^−03^ belonging to the thrombin signalling pathway. Gene symbol, gene name as reported in the Entrez database, the dbSNP variant ID, the *P* value of the aforementioned variant in the multivariate analysis, the location and type of the associated protein are reported here

After stepwise regression including all significant non-genetic covariates as well as rs9923231, rs1057910 and rs1799853, ex-smoker status and alcohol consumption were not retained in the final model. Percentage of the MWD variance explained in the complete model was 57.4 % (breakdown was age 11.20 %, height 3.56 %, weight 5.98 %, follow-up time 1.20 %, number of cigarettes smoked/day 3.12 %, interacting co-medications 0.98 %, sum of effects of interacting medications 2.20 %, non-interacting medications 0.61 %, rs9923231 25.61 %, rs1057910 12.93 % and rs1799853 3.72 %). Inclusion of rs2108622 (CYP4F2) brought the total variation explained to 57.89 %.

In the 160 patients for which baseline blood count and coagulation factor levels were available, age, height, weight, haemoglobin, basophils, urea, ALT, factor IX and smoking status were found significant in the univariate regression on MWD, and included as covariates in the multiple regression models. As expected given the low power, only rs9923231 (VKORC1) reached genome-wide significance, as well as an imputed SNP in the CYP2C9 locus (rs1072753, R^2^ = 0.61 with rs1057910, CYP2C9*3, in 1000 genome project pilot 1) (Additional file 3). CYP2C9*3, instead of the imputed rs1072753, was used in the stepwise regression. Only age (13.39 %), weight (7.58 %), Factor IX (4.37 %), smoking status (9.98 %), rs9923231 (18.15 %) and rs1057910 (14.97 %) were retained in the final model, explaining a total of 59.64 % of the MWD variation.

**Fig. 2:**
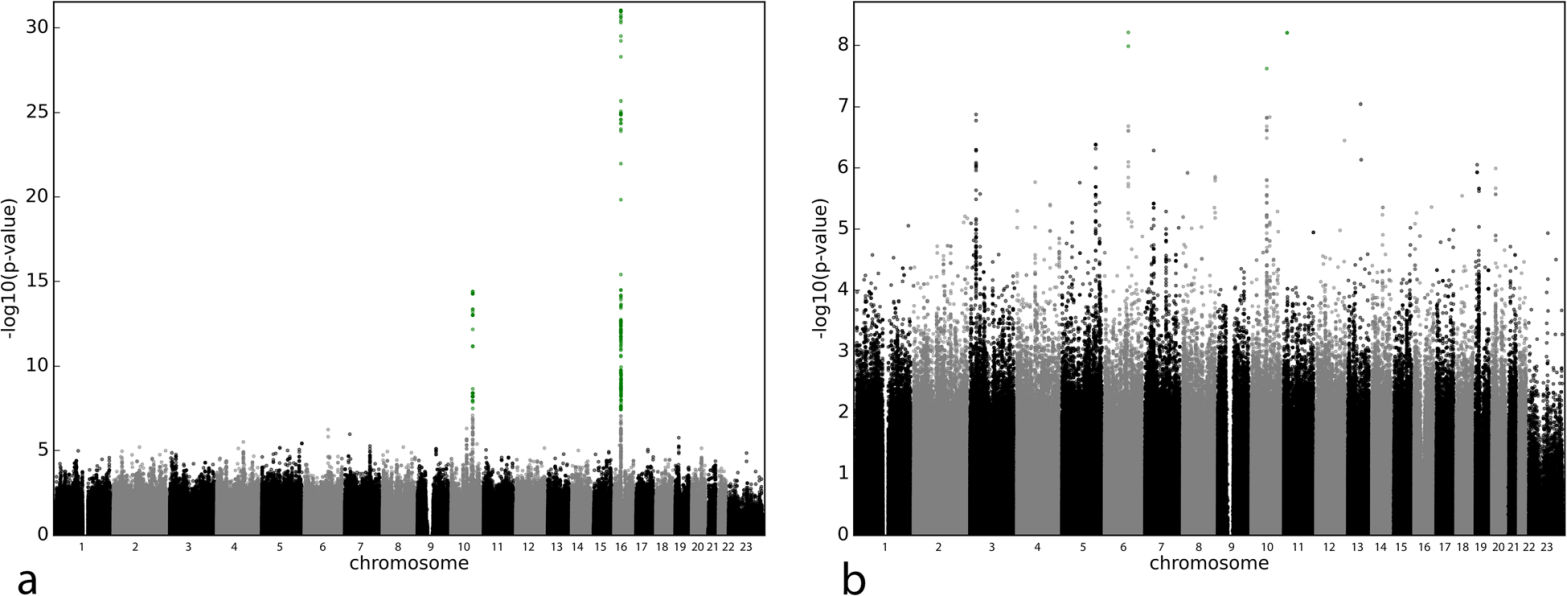
Manhattan plots for multiple regressions on SMWD only adjusting for non-genetic factors (**a**) and adjusting for non-genetic and genetic factors (**b**). The vertical axis represents the common logarithm of the *P* value, numbers on the horizontal axis represent the chromosome. Signals below the genome-wide significance threshold of 5 × 10^−08^ are represented in green

For warfarin SMWD (Additional file 1 Sheet 3), the univariate signals (Fig. 2a) were rs9923231 (*P* = 9.26 × 10^−32^) for VKORC1, and rs4917639 (composite CYP2C9 *2/*3, *P* = 5.26 × 10^−15^) and rs1057910 (CYP2C9*3, *P* = 3.95 × 10^−09^) for CYP2C9, while rs1799853 (CYP2C9*2, *P* = 4.70 × 10^−07^) did not reach genome-wide significance. Despite not reaching genome-wide significance, rs1799853 was included in the multiple regression as it influences warfarin dose, along with rs9923231 and rs1057910. Three signals reached genome-wide significance after adjusting for genetic and non-genetic factors (Fig. 2b): 6–106244024 (*P* = 6.15 × 10^−09^), 11–15383178 (*P* = 6.23 × 10^−09^) and rs112942398 (*P* = 2.40 × 10^−08^). All these SNPs are the result of imputation; there is close to no support from surrounding genotyped SNPs for 11–15383178 and 6–106244024 (data not shown), unlike for rs112942398 (Additional file 4), which is gene-rich, but none of the genes had a link to warfarin metabolism or blood coagulation. To further assess the validity of rs112942398, 94 samples were genotyped using a KASPar custom assay; 10 of the aforementioned samples were given as homozygous for the minor allele by the imputation, and 42 were given as heterozygous, the remainder being given as homozygous for the major allele. Four samples failed genotyping, leaving 90 genotypes available for comparison. There were 12 discordant genotypes between imputation and KASPar genotyping, giving an approximate imputation error rate of 13.3 %.

All covariates from the multiple regression remained in the final model after stepwise regression, with age explaining 9.72 % of SMWD variation, height 4.45 %, weight 7.94 %, interacting co-medications 1.92 %, rs9923231 30.38 %, rs1057910 8.08 % and rs1799853 3.81 %. The final model explained 55.27 % of the variation. Inclusion of rs2108622, retained after stepwise regression, brought the total variation explained to 56.97 %.

### GWAS analysis for INR >4.0 in the first week

**Fig. 3:**
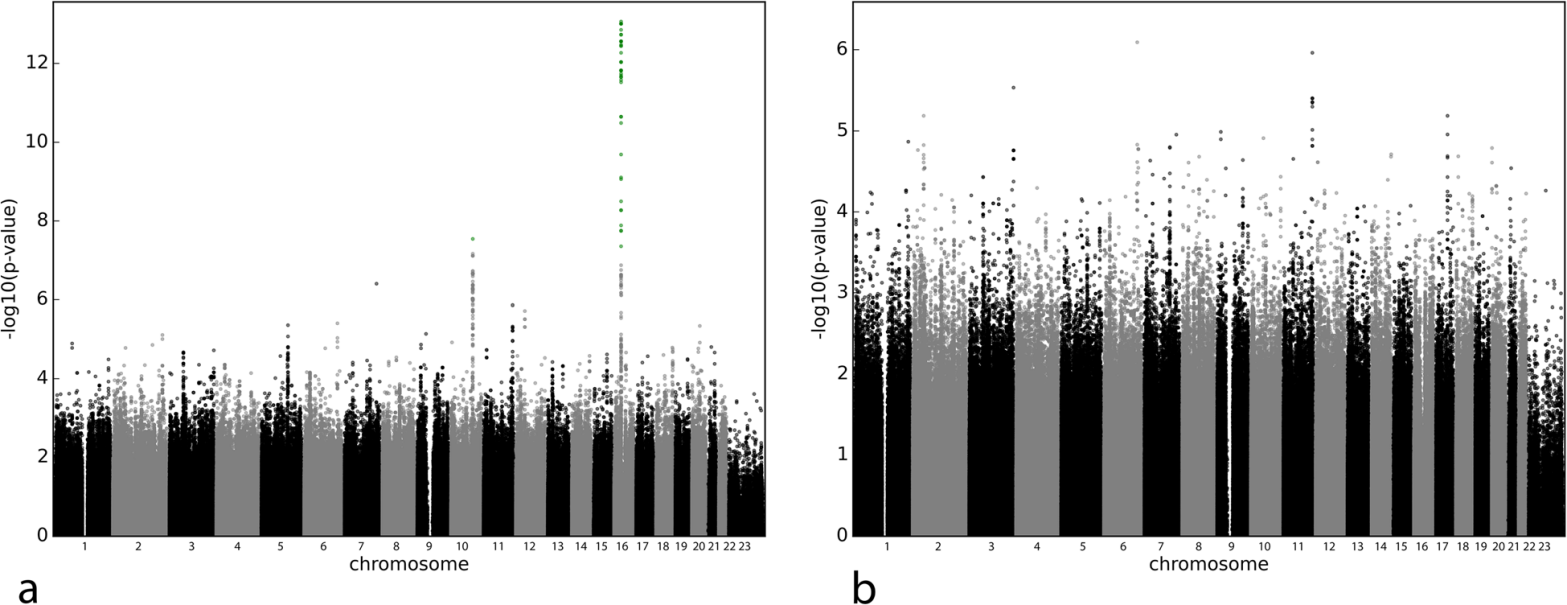
Manhattan plots for multiple regressions on INR >4 during first week of treatment only adjusting for non-genetic factors (**a**) and adjusting for non-genetic and genetic factors (**b**). The vertical axis represents the common logarithm of the *P* value, numbers on the horizontal axis represent the chromosome. Signals below the genome-wide significance threshold of 5 × 10^−08^ are represented in green

Age, height, weight, loading dose, alcohol consumption and medications not known to interact with warfarin were significant in the univariate analysis and were adjusted for when testing for SNP associations. Two regions harbour signals reaching genome-wide significance (Additional file 1 Sheet 4 and Fig. 3a): the lowest *P* value was observed for rs2288004 (*P* = 8.76 × 10^−14^) on chromosome 16, an imputed SNP in high LD with VKORC1’s rs9923231 (R^2^ = 0.98), followed by rs1072753 (*P* = 2.85 × 10^−08^), an imputed SNP on chromosome 10 in high LD with CYP2C9*3 (rs1057910, R^2^ = 0.78). After accounting for rs2288004 and rs1072753 and non-genetic factors, no other signal reached genome-wide significance (Fig. 3b). Individuals homozygous for rs9923231 minor allele had an odds ratio of 8.04 (4.43–14.90, N = 83) for having an INR >4.0 during the first week of treatment, while the odds ratio for heterozygous patients was 2.22 (1.34–3.78, N = 287), in comparison to patients homozygous for the major allele (N = 255); similarly, the odds ratio for patients homozygous for rs1057910 minor allele was 24.17 (3.66–644.29, N = 6) and 2.93 (1.70–4.96, N = 74) for heterozygous, reported against homozygous patients for the major allele (N = 545). Two signals not quite reaching genome-wide significance appear in regions of biological relevance to clotting; rs747180 (*P* = 1.09 × 10^−06^) is located in an intron in APLP2, which has been linked to haemostasis through its inhibitory effect on Factor XIa [24, 25], and rs6809892 (*P* = 2.94 × 10^−06^) is located near TFRC, a gene implicated in the development of erythrocytes [26].

### Sensitivity analyses

There were 33 patients with at least one clinical value over three standard deviations from the cohort mean. We repeated all the regression analyses after excluding these outlier patients; this did not significantly alter any of our findings (data not shown).

## Discussion

Non-genetic factors [27] and two main genetic factors [21, 28–32] influence warfarin stable dose, with about 50 % of the variance remaining unexplained. Studies have mainly focused on stable dose, neglecting factors influencing the initial response to warfarin therapy, a period during which patients are at high risk of over- or under-anticoagulation [8, 14]. Our prospective study of 714 British patients undergoing warfarin therapy, from initiation to a 6-month follow-up, has the advantage of being able to capture clinical parameters difficult to assess through a retrospective study design, as well as monitoring a wider range of outcomes, such as patient response at the time of therapy initiation. As this is a prospective cohort, and to be true to the diversity of medical conditions encountered in the clinic, no patients were excluded based on their co-morbidities, no matter how severe. However, removing patients with outlier values for clinical data does not significantly change the results of the regressions.

For both MWD and SMWD, we confirm previous findings that *VKORC1* and CYP2C9 *2 and *3 are the major genetic determinants of warfarin dose [21, 28–32]. *VKORC1 rs9923231* explained between 25.6 % and 30.4 % of the dose variance (MWD and SMWD, respectively), CYP2C9*3 between 8.1 % and 12.9 %, and CYP2C9*2 between 3.7 % and 3.8 %, all in accordance with previous findings [32–37]. Unlike in some previous studies [22, 23] *CYP4F2 rs2108622* did not reach genome-wide significance, but retaining it in the model explained 0.5 % of MWD and 1.7 % of SMWD. No other variants reached genome-wide significance in the MWD analysis. Pathway analysis of the top variants post-conditioning for MWD implicated multiple hits in the thrombin signalling pathway, but none of these variants remained in the model after stepwise regression, suggesting that they had little influence on the overall MWD. For SMWD, three variants remained significant after conditioning: while two of them are most probably imputation artefacts, the third signal, *rs112942398*, looks like a much more plausible signal based on its regional plot. However, this is likely to be an artefact given that its biological relevance is unclear, it has not been picked up by other studies [23, 34], and imputation error rate was high. These data confirm that other genetic factors beyond *CYP2C9* and *VKORC1* are unlikely to make a strong contribution towards the variance in warfarin dose.

In terms of clinical factors for SMWD, these were similar to those previously described in the literature [14, 27, 38, 39], namely age, height, weight and use of interacting co-medications. For MWD, we were able to explore some novel parameters including the follow-up period, which explained up to 1.2 % of the variance in dose, demonstrating the importance of an extended follow-up period on mean weekly dose calculations. Interacting co-medications explained 1 % of the dose variance. Since patients on warfarin are on multiple medications, which may all potentially interact with warfarin, sometimes with opposite effects, we evaluated the sum of these interactions which explained up to 2.2 % of the variance, showing that much greater effect of co-medications can be determined by taking into account the effect of all of them. The number of cigarettes smoked per day explained up to 3.2 % of the variance, similar to that seen by co-medications. It is possible that this effect is mediated through the induction of CYP1A2 by cigarette smoke [40], which metabolises R-warfarin.

In the subset of patients with extended clinical data, for the MWD, two clinical factors, on top of age and weight, were of importance and retained after stepwise regression: (a) smoking, rather than the more refined number of cigarettes per day, was highly significant and explained a large portion of the variance at about 10 %; and (b) baseline Factor IX levels explained approximately 4.4 % of the MWD variance, far more than the 0.5 % explained by *rs2108622* (*CYP4F2*). The impact of warfarin on the various clotting factor levels, and how quickly they respond to warfarin, is not clear, and needs further investigation.

For INR >4.0 during the first week of treatment, only two genetic factors were important: *VKORC1 rs9923231*, with odds ratios of 7.9 and 2.23, for homozygous for the minor allele and heterozygous patients, respectively, and CYP2C9*3, with odds ratios of 24.22 and 2.88, respectively. The warfarin loading dose also seems to play an important role in this outcome measure with a univariate *P* value of 5.9 × 10^−07^ – within our cohort there was variability in loading doses used, ranging from 3 to 32 mg (median: 21 mg, mean: 19.42 mg), adding to the inter-patient variability in warfarin response. Taken together, our data indicate the importance of taking *VKORC1* and *CYP2C9* genotype into account when determining the loading dose, consistent with our loading dose algorithm [41], which when tested in the EU-PACT trial [11] reduced the risk of patients in the genotype-guided dosing arm having an INR >4.0. Interestingly, known interacting co-medications did not show a significant effect on the outcome INR >4.0, while other co-medications did. The reason for the latter is unclear, as there were medications from many different therapeutic classes present, and thus the most likely explanation is that this is a surrogate for co-morbidities affecting anticoagulation response. This is consistent with a recent cross-sectional study from France which showed that comorbidities worsened the quality of INR control [42]. In patients with extended baseline data, triglycerides and factor VII levels also affected the risk of INR >4.0. Factor VII, a vitamin K-dependent clotting factor, was highly correlated to factor IX. There is lack of knowledge concerning the inter-individual variation in Factor VII levels in response to warfarin. Unfortunately, replication of these results remains impossible at the moment, as no other warfarin study has recorded such a wide range of clinical factors, and/or measured baseline clotting factor levels. Furthermore, given the high cost involved in measuring clotting factor levels, their use in clinic is unlikely. The regulation of clotting factors is most probably complex and the genetic variants involved in such processes are likely to have a low to moderate impact on warfarin response (below 5 % for mean dosing), and thus are unlikely to be included in dosing algorithms unless whole genome sequencing data become incorporated into patient records. Curiously, while ALT was found to contribute significantly to over-anticoagulation during warfarin initiation in a cohort of patients from Asian descent, it was not significant in our INR >4 analysis [43]. It was found to be significantly associated with MWD in our univariate regressions, but was not retained in the final model. On the other hand, it is interesting to note that APOE *ε4 was associated with lower warfarin dose in a cohort of Brazilian patients [44], possibly echoing our finding about the influence of triglycerides on over-anticoagulation. Unfortunately, rs429358, one of the two variants, with rs7412, used to code this APOE allele, was neither genotyped not imputed in our cohort, not allowing us to investigate further if this allele was linked to our findings.

## Conclusions

In conclusion, our analysis shows that multiple factors, genetic and clinical, are important in determining the response to warfarin, which is perhaps not surprising given the pharmacology of warfarin. *VKORC1* (*rs9923231*), CYP2C9 *3 and *2 are the most important genetic factors influencing warfarin dose, with *CYP4F2* (*rs2108622*) having a minor effect, with age and BMI being important clinical covariates. Patients’ smoking habits and the totality of interacting co-medications, however, also seems to be important when determining warfarin dose. In relation to INR >4 after warfarin initiation, *VKORC1* and CYP2C9*3 are important in determining the loading dose, together with alcohol consumption. Realistically, at the present time, it would not be possible to evaluate each of the clinical factors in trials to optimise warfarin dosing. Furthermore, in a randomised design, confounding clinical factors are likely to be balanced between two arms. Thus, the trials which have been undertaken, which take into account *CYP2C9* and *VKORC1* genotype, together with age and BMI in determining dosing algorithms, represent pragmatic designs in a Northern European population.

## Additional files

Additional file 1: Sheet 1. Interaction coefficients of drugs known to be interacting with warfarin. Drugs known to interact with warfarin were classified according to their effect on warfarin dosing, with a plus sign for potentiating drugs, and minus for the opposite effect, the absolute value representing the scale of the effect. When several of these drugs were used concomitantly the sum of these coefficients was used. **Sheet 2.** List of all concomitant drugs taken by patients. **Sheet 3.** Top signals from linear regressions on SMWD. *P* values marked with an asterisk were obtained from the regression only adjusting for non-genetic factors, while other *P* values where obtained from a multiple regression after conditioning on rs9923231, rs1799853 and rs1057910. **Sheet 4.** Top signals from logistic regressions on INR >4 during first week of treatment. *P* values marked with an asterisk were obtained from the regression only adjusting for non-genetic factors, while other *P* values where obtained from a multiple regression after conditioning on rs2288004 and rs1072753. *P* values for rs9923231, CYP2C9 *2 and *3 are reported, albeit they were not the most statistically significant signals in their respective region. 95 % confidence intervals for odds ratios are indicated in parentheses. (XLSX 28 kb) Additional file 2:
**The CNV QC and analysis method.** (DOCX 39 kb) Additional file 3:
**Manhattan plot for a multiple regression on MWD with additional non-genetic variables.** The vertical axis represents the common logarithm of the *P* value, numbers on the horizontal axis represent the chromosome. Signals below the genome-wide significance threshold of 10^−08^ are represented in green. In addition to age, height and weight, Factor IX levels, smoking status, ALT, urea levels, haemoglobin count and basophils count were used as covariates in the regression on warfarin mean dose. (PNG 1601 kb) Additional file 4:
**Regional plot of signals around rs112942398. The left vertical axis represents the absolute value of decimal logarithm of the** ***P*** **value, the right vertical axis represents the recombination rate, in centimorgans per megabase, and the horizontal axis represents the position, in base pairs, along chromosome 10, according to the NCBI 36 reference.** The colour associated with each signal represents the amount of linkage disequilibrium with the main signal in the region, the latter being represented on the plot by a purple diamond; the colour coding is explained in the legend box on the right of the figure. Genes in the regions are represented by arrows which indicate their approximate position, length and transcription direction, the arrow head pointing toward 3’. (PNG 1288 kb)

## Abbreviations

ALT: Alanine Transaminase
BMI: Body Mass Index
FDA: Food and Drug Administration
INR: International Normalized Ratio
MAF: Minor Allele Frequency
MWD: Mean Weekly Dose
SMWD: Stable Mean Weekly Dose
SNP: Single Nucleotide Polymorphism
