# Supplementary material for: A multi-factorial analysis of response to warfarin in a UK prospective cohort
Source: Genome Med. 2016 Jan 6;8:2. doi: 10.1186/s13073-015-0255-y (PMC4702374; doi:10.1186/s13073-015-0255-y)
Supplement: Additional file 2: — The CNV QC and analysis method. (DOCX 39 kb) [file 13073_2015_255_MOESM2_ESM.docx]

**Genome-wide association study of response to warfarin in a UK prospective cohort**

**Supplementary Material & Methods**

**Imputation**

Following sample QC, an impute-ready file was generated after filtering the data as follows, using PLINK v1.07[[1](#_ENREF_1)] and R v2.11.1[[2](#_ENREF_2)]:

1. SNPs

- MAF>0.01
- HWE exact p >0.0001
- Call rate >0.95 if MAF>0.05
- call rate >0.98 if MAF between 0.03 and 0.05
- call rate >0.99 if MAF between 0.01 and 0.03
- remove sex-linked SNPs

1. Samples

- Samples with a remaining overall genotyping rate below 95% after removal of SNPs failing the aforementioned criteria were excluded
- Samples with overall heterozygosity further than three standard deviations from the mean were excluded
- Samples with ambiguous gender were excluded
- Removal of ethnic outliers after Principal Component Analysis (PCA) using smartPCA [[3](#_ENREF_3)] with HapMap 3 cohorts, as well as patients self declared of mixed or non-caucasian ancestry.

Imputation of genotypes was carried out using IMPUTE V2.1 [[4](#_ENREF_4)], with the filtered combined set of HapMap 3 release 2 (Feb 2009) and 1000 genomes pilot 1 CEU (March 2010) [[5](#_ENREF_5)]. In order not to bias genotyping against heterozygous positions, imputation quality metrics from IMPUTE were used instead of the usual 0.9 threshold on genotype uncertainty; SNPs with an information measure below 0.8 were discarded using QCtool [http://www.well.ox.ac.uk/~gav/qctool/ ], and a threshold of 0.5 was applied on genotype uncertainty using Gtool [http://www.well.ox.ac.uk/~cfreeman/software/gwas/gtool.html]. Sex-linked SNPs were added back to the imputed genotype set, and filtering was carried out again with the same settings as earlier, with the following exceptions: SNPs were not excluded based on HWE exact p-value, sex-linked SNPs were not excluded, no further PCA analysis was carried out to identify ethnic outliers.

**CNV calling and QC**

CNVtools does not attempt CNV discovery; instead, it uses a predefined list of known CNV regions. The list used was that published by Conrad *et al*. [[6](#_ENREF_6)]. Only the 11,306 autosomal regions listed were considered. Of these, 5,972 had at least one Illumina probe present and so were run through the CNV analysis pipeline. This was performed for the continuous (quantitative) traits √(Mean Weekly Dose), *SQRTMWD*, and √(Stable Mean Weekly Dose), *SQRTSMWD*, in turn. At the start of the CNV analysis there were, respectively, 635 and 332 samples with valid (non-NA) values of these traits.

For a given (predefined) CNV region, samples with too many NA values were removed; CNVtools then performed a principal component analysis (PCA) on a matrix of standardised signal intensities and clustered the result of the PCA; the clusters represented the copy number assignments for the samples. Output from the PCA procedure could then be used in linear discriminant analysis (LDF) to further improve the clustering of the data [[6](#_ENREF_6)].

The approach taken (post sample QC) was: (i) initial QC, (ii) first fitting attempts for all regions (“run 1”), (iii) further QC, (iv) second and final fitting attempts (“run 2”), (v) identification of “interesting” regions, (vi) additional checks and (vii) visual inspection of the clusters produced by CNVtools and comparison of these with the observed intensity signal distribution.

Detail of the approach taken post sample QC:

1. *Initial QC*. This was done on a per-chromosome basis. The value of the Illumina BeadStudio quantity *R=X+Y*, i.e. the total BeadStudio-normalised intensity[[7](#_ENREF_7)], was found for each sample at each probe position. It was assumed that *R* was a proxy for the underlying copy number. After removing NaN values, two quantities were calculated for each sample: (a) the median value of *R*, and (b) *mad.d1r*, the median of absolute differences in copy number measurement between neighbouring probes. Scatter plots of the two parameters and subsequent visual inspection aided cut-off values to be determined for sample rejection; 3 standard deviations (s.d.) from the mean were used in this case. Extreme (> 3 and < 3 s.d.) values of median *R* indicated possible aneuploidies (in practice, probably cell-line artefacts), whereas values of *mad.d1r* > 3 s.d. above the mean cast doubt on the veracity of the probe readings, as this indicated frequent sudden changes in *R* between adjacent probes. Samples with values outside the bounds described were deemed to have failed initial QC for, and were removed from the analysis of, that chromosome. This process was repeated for each chromosome in turn.
2. *Run1*. CNVtools has parameters which can be specified to influence the way it attempts its cluster fitting. The parameters considered were: whether to use Gaussian or *t* distributions; whether to force a 3-cluster model or allow CNVtools to estimate the number of clusters freely; and whether to allow each cluster’s variance to be different or force them to be equal. There were thus 8 (=2^3^) possible parameter combinations.

Fits were attempted for all regions, with at least one probe present, for each chromosome in turn; samples which had failed initial QC for a given chromosome were not included in the fitting attempts for that chromosome’s regions. “Pass” fits were deemed to be those which converged at all three fitting stages and had values of the cluster separation parameter *Q* > 4 [[8](#_ENREF_8)]. An automatic refitting procedure was implemented which tried all 8 parameter combinations in turn, stopping if a fit had passed. At the end of this process not all CNV regions achieved a pass fit; some had lower *Q* values, some did not converge at all three stages and some ran into difficulties, namely posterior probability problems or software errors.

1. *Further QC*. This step identified samples which tended to lie in the tails of the model clusters, i.e. less confidence could be attached to the assigned cluster numbers of these samples. For each region, CNVtools recorded the mean and standard deviation of each cluster it deduced during the fitting process, together with which cluster each sample was assigned to. Regions which did not run into difficulties (including those with *Q* ≤ 4) were used for further QC. For each such region, the means and standard deviations of the fitted clusters, together with each sample’s cluster number and signal value, were noted. In cases of uncertainty (e.g. a sample lying near the midpoint of a region overlapped by the tails of two adjacent clusters) the fit assigned a cluster number of -1 to the sample; otherwise, the number of valid regions for a given sample was incremented by 1. Let *N* be the number of regions, in a given chromosome, processed in this way. Let *n* be the number of valid regions obtained for a particular sample. Samples with *n* < 0.95*N* were rejected.

For each remaining sample, a metric

$$\bar{S}=\frac{\sum_{i=1}^{n} \frac{\bar{x}-x_{i}}{\sigma_{cluster i}}}{n}$$

was calculated across its *n* valid regions, where σ*_cluster i_* was the standard deviation of the cluster to which the sample had been assigned for the *i*^th^ CNV region, $\bar{x}$ the mean of that cluster and *x_i_* the sample’s signal value. The distribution of ln($\bar{S}$) was approximately normal, and a boxplot approach was used to identify high outliers from this distribution; a sample was excluded if its ln($\bar{S}$) value was more than 1½ times the inter-quartile range above the third quartile[[9](#_ENREF_9)].

1. *Run 2*. Having removed the “persistent outlier” samples found in the further QC step, the automatic refitting approach was again used to maximise the number of pass fits.
2. *Identification of “interesting” regions*. CNVtools produced posterior probability files for fitted regions under both H_0_ (no correlation between copy number and the quantitative trait under investigation) and H_1_ (correlation). The χ^2^ goodness-of-fit value was extracted from each region which had passed the run 2 fitting process; this was defined[[9](#_ENREF_9)] as χ^2^ = - 2ln(*L_0_*/*L_1_*), *L_0_* and *L_1_* being the likelihood of the fit under H_0_ and H_1_, respectively. The CNVtools function qq.chisq() (from the “Genotools” part of the package) was used to produce a QQ plot of the observed *vs.* expected χ^2^ distributions. Regions lying outside the plot’s 95% probability region, and having χ^2^ > 10, were noted for further investigation.
3. *Additional checks*. The posterior probability files were checked for all fitting attempts. CNV regions were marked for exclusion if the fitted model had inconsistent cluster numbering (e.g. claiming to have clusters numbered 1, 2, 4 and 5 but no samples in cluster 3). Any regions in the “further investigation” list obtained in the previous step were removed from that list if they failed this check.
4. *Plot inspection*. Finally, the remaining regions in the “further investigation” list were plotted using a variant of the CNVtools cnv.plot() function. These showed histograms of the input intensity signals, having undergone the PCA (and sometimes LDF) and standardisation steps; posterior probability (fitted cluster estimate) lines were overlaid on these. Visual inspection was then used to discard fits which were obviously wrong, i.e. the fit was not a good match to the intensity histogram.

REFERENCES

1. Purcell S, Neale B, Todd-Brown K, Thomas L, Ferreira MA, Bender D et al. PLINK: a tool set for whole-genome association and population-based linkage analyses. Am J Hum Genet. 2007;81(3):559-75. doi:S0002-9297(07)61352-4 [pii]

10.1086/519795.

2. R Development Core Team R. R: A language and environment for statistical computing. R Foundation forStatistical C om puting, V ienna, A ustria, ISB. 2006:07-0.

3. Patterson N, Price AL, Reich D. Population structure and eigenanalysis. PLoS Genet. 2006;2(12):e190. doi:06-PLGE-RA-0101R3 [pii]

10.1371/journal.pgen.0020190.

4. Howie BN, Donnelly P, Marchini J. A flexible and accurate genotype imputation method for the next generation of genome-wide association studies. PLoS Genet. 2009;5(6):e1000529. doi:10.1371/journal.pgen.1000529.

5. Marchini J, Howie B. Genotype imputation for genome-wide association studies. Nat Rev Genet. 2010;11(7):499-511. doi:nrg2796 [pii]

10.1038/nrg2796.

6. Conrad DF, Pinto D, Redon R, Feuk L, Gokcumen O, Zhang Y et al. Origins and functional impact of copy number variation in the human genome. Nature. 2010;464(7289):704-12. doi:nature08516 [pii]

10.1038/nature08516.

7. Illumina. Genotyping Data Normalization Methods. 2007.

8. Conrad DF, Pinto D, Redon R, Feuk L, Gokcumen O, Zhang Y et al. Origins and functional impact of copy number variation in the human genome. Nature. 2010;464(7289):704-12. doi:10.1038/nature08516.

9. NIST/SEMATECH. e-Handbook of Statistical Methods. <http://www.itl.nist.gov/div898/handbook/eda/section3/boxplot.htm> and <http://www.itl.nist.gov/div898/handbook/apr/section2/apr233.htm>. Accessed September 2009.
