# Supplementary figures and images for: A multi-factorial analysis of response to warfarin in a UK prospective cohort
Source: Genome Med. 2016 Jan 6;8:2. doi: 10.1186/s13073-015-0255-y (PMC4702374; doi:10.1186/s13073-015-0255-y)

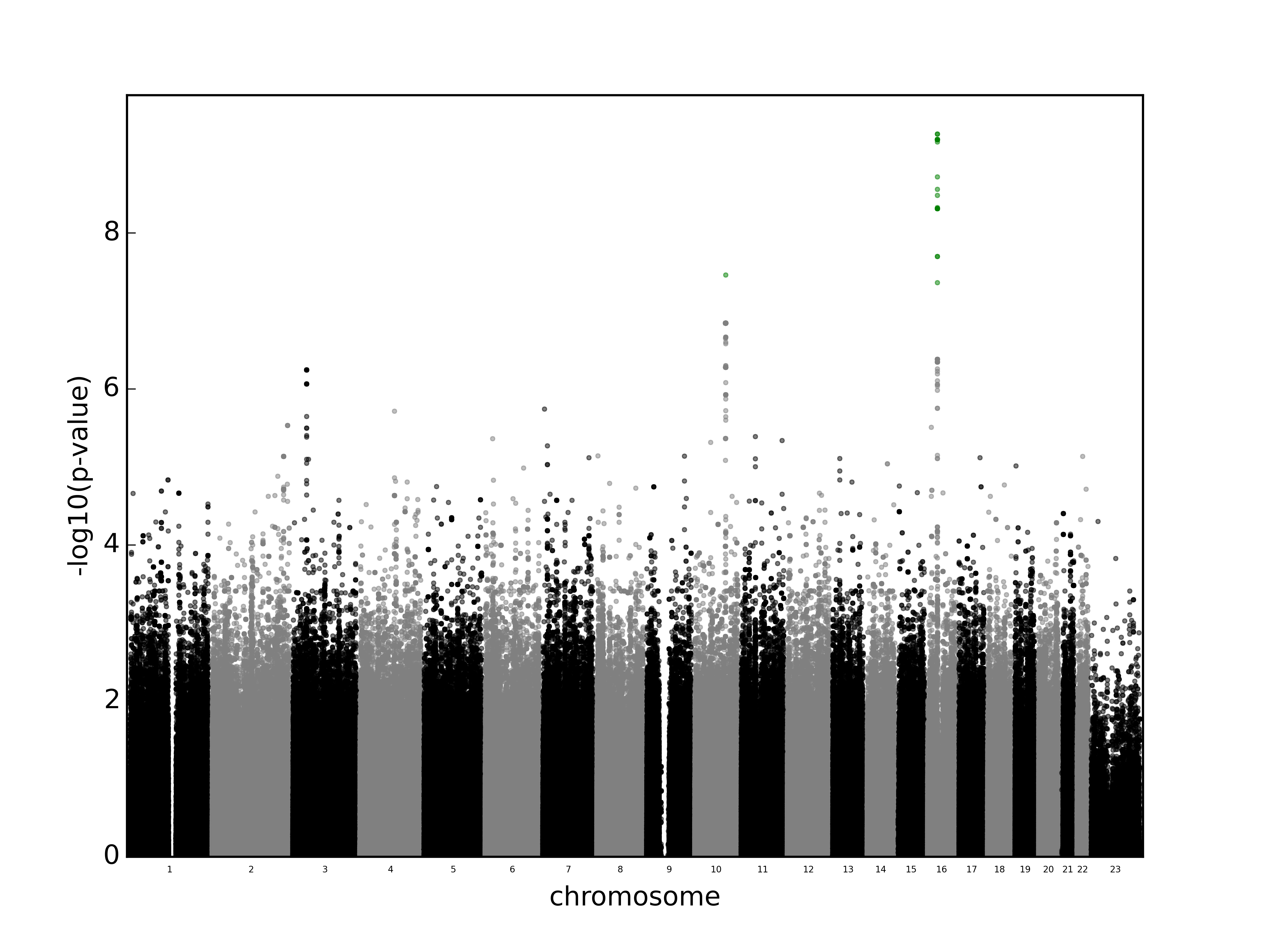

Supplement: Additional file 3: — Manhattan plot for a multiple regression on MWD with additional non-genetic variables. The vertical axis represents the common logarithm of the P value, numbers on the horizontal axis represent the chromosome. Signals below the genome-wide significance threshold of 10−08 are represented in green. In addition to age, height and weight, Factor IX levels, smoking status, ALT, urea levels, haemoglobin count and basophils count were used as covariates in the regression on warfarin mean dose. (PNG 1601 kb) [file 13073_2015_255_MOESM3_ESM.png]

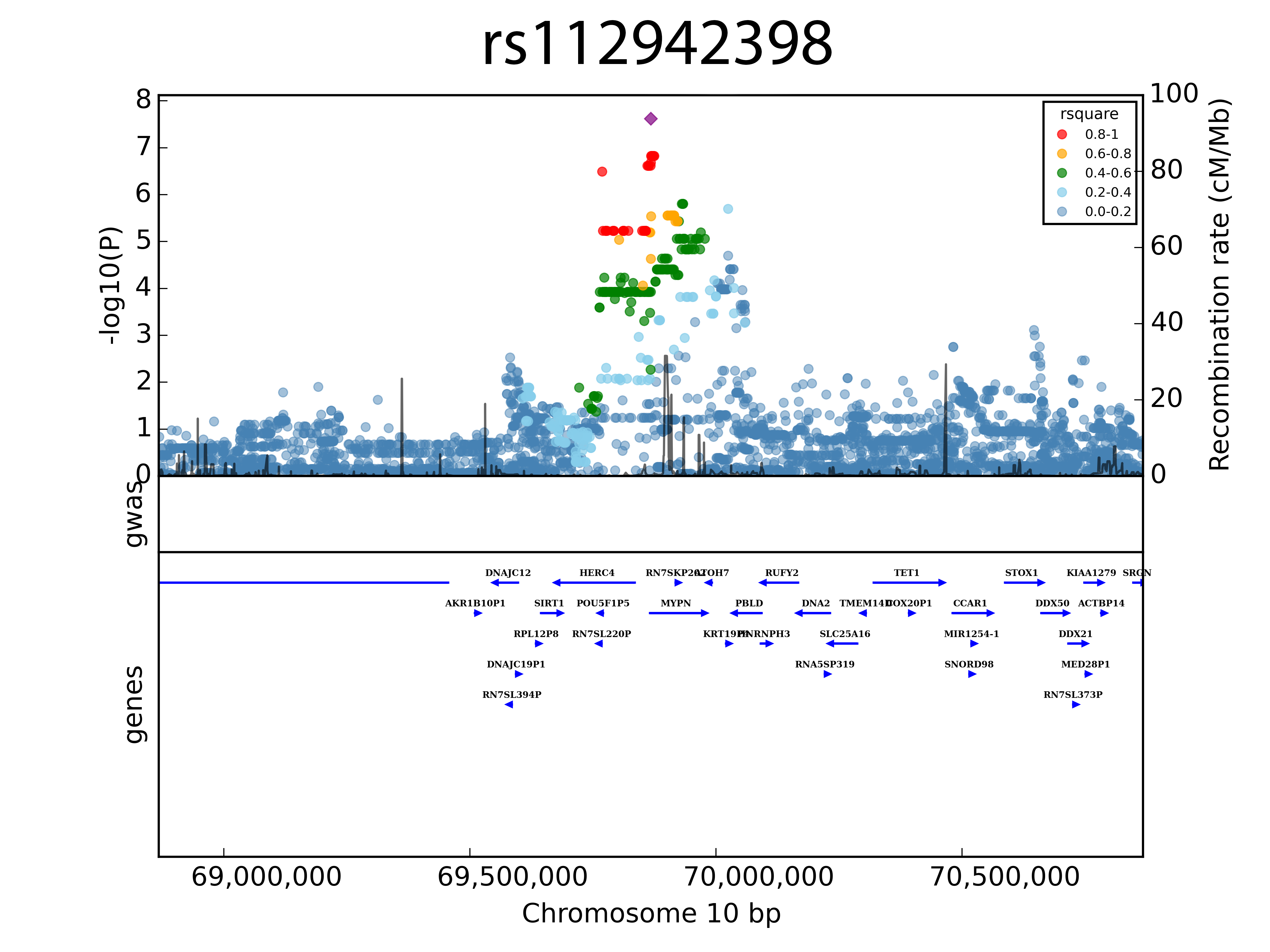

Supplement: Additional file 4: — Regional plot of signals around rs112942398. The left vertical axis represents the absolute value of decimal logarithm of the P value, the right vertical axis represents the recombination rate, in centimorgans per megabase, and the horizontal axis represents the position, in base pairs, along chromosome 10, according to the NCBI 36 reference. The colour associated with each signal represents the amount of linkage disequilibrium with the main signal in the region, the latter being represented on the plot by a purple diamond; the colour coding is explained in the legend box on the right of the figure. Genes in the regions are represented by arrows which indicate their approximate position, length and transcription direction, the arrow head pointing toward 3’. (PNG 1288 kb) [file 13073_2015_255_MOESM4_ESM.png]
